# Deceleration Capacity as a Marker of Autonomic Cardiac Modulation in Prodromal and Manifest Parkinson's Disease, Multiple System Atrophy, and Progressive Supranuclear Palsy

**DOI:** 10.1111/ene.70395

**Published:** 2025-10-30

**Authors:** Elisabeth Ruppert, Nuria Mix, Karl Kesper, Axel Bauer, David Vadasz, Vincent Ries, Elisabeth Sittig, Ulrich Koehler, Annette Janzen, Wolfgang H. Oertel

**Affiliations:** ^1^ Department of Neurology Philipps University of Marburg Marburg Germany; ^2^ Department of Neurology, CIRCSom (International Research Center for ChronoSomnology) & Sleep Disorders Center University Hospital of Strasbourg, University of Strasbourg Paris France; ^3^ Department of Pulmonology University of Marburg Marburg Germany; ^4^ Department of Cardiology and Angiology, Clinic of Internal Medicine University Hospital Innsbruck Innsbruck Austria

**Keywords:** atypical parkinsonian syndromes, deceleration capacity, heart rate variability, isolated REM sleep behavior disorder, Parkinsons disease

## Abstract

**Background:**

Degenerative parkinsonian syndromes, including the alpha‐synucleinopathies (aSYN) Parkinson's disease (PD), and multiple system atrophy (MSA), and the tauopathy progressive supranuclear palsy (PSP), are characterized by motor and non‐motor symptoms. The later subsume autonomic dysfunction, which may appear early or progress with the disease. Cardiac dysfunction varies by syndrome and can also occur in isolated REM sleep behavior disorder (iRBD), a prodromal stage of aSYN. Overlapping motor features make early differentiation challenging. Heart rate variability (HRV) analysis is a noninvasive tool for evaluating cardiac autonomic function, with deceleration capacity (DC) as a sensitive parasympathetic marker. This study compares HRV and DC across parkinsonian syndromes to assess their potential in early diagnosis and differentiation.

**Methods:**

Using standardized 30‐min resting ECG recordings in the early morning, we analyzed HRV parameters in five groups: iRBD (*n* = 10), PD (*n* = 10), MSA (*n* = 10), PSP (*n* = 9), and healthy controls (HC, *n* = 10). Evaluated HRV parameters included HRV index (HRVI), reflecting overall variability, and DC.

**Results:**

As expected, DC was significantly lower in MSA (3.82 ± 1.38) and unexpectedly even lower in PSP (3.19 ± 2.77), compared to HC (9.66 ± 4.67) and PD (7.55 ± 2.48). These findings are novel for PSP. HRVI was significantly reduced in PSP, while other HRV parameters showed no significant differences.

**Conclusions:**

Deceleration capacity (DC) reduction in MSA and PSP suggests pronounced cardiac parasympathetic dysfunction. DC may support differentiation between PD and atypical syndromes, but larger studies are needed for validation. Given the impact of autonomic dysfunction on quality of life and mortality, comprehensive autonomic testing should be included in the diagnostic workup.

## Graphical abstract

Objective: Heart rate variability (HRV) is a promising tool for autonomic dysfunction assessment, especially Deceleration capacity (DC), a sensitive parasympathetic marker. M&M: Study in patients with isolated REM sleep behavior disorder, Parkinson's disease, multiple system atrophy, and supranuclear palsy as compared to healthy control subjects. Results: Declaration capacity was reduced in MSA and in PSP, indicating cardiac parasympathetic dysfunction. Reduced HRV index reflects overall impaired cardiac autonomic adaptability in PSP. DC may help differentiate Parkinson's disease from MSA and PSP.

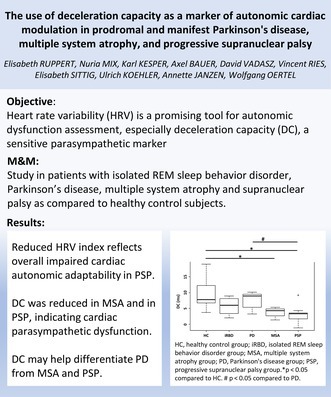

## 1 Introduction

Degenerative parkinsonian syndromes, including Parkinson's disease (PD), multiple system atrophy (MSA), and progressive supranuclear palsy (PSP), have motor symptoms such as akinesia and rigidity in common. PD and MSA are alpha‐synucleinopathies (aSYN), whereas PSP is a tauopathy. Beyond motor impairment, these disorders present with diverse non‐motor symptoms that significantly affect quality of life. Non‐motor symptoms may precede motor onset, emerge early, or develop in later disease stages, suggesting their potential as early diagnostic markers [2, 3, 24]. Autonomic dysfunction is a key feature, varying by disease, and includes orthostatic dysregulation, impaired thermoregulation, and genito‐urethral, intestinal, and cardiac dysfunction [4]. In isolated REM sleep behavior disorder (iRBD), considered a prodromal stage of aSYN such as PD and MSA, autonomic dysfunction can occur in the absence of motor symptoms [5, 6].

Cardiac dysfunction in these disorders results from impaired heart innervation, driven by disease‐specific mechanisms. In PD, autonomic degeneration can occur early [7, 24]. Respective myocardial innervation studies using metaiodobenzylguanidine (MIBG) scintigraphy reveal postganglionic sympathetic denervation in initial disease stages [7, 8], though some early‐stage patients may show normal findings [8]. MIBG alterations correlate with disease progression, as represented by worsening Hoehn and Yahr stages [8]. Similar postganglionic sympathetic degeneration is observed in iRBD, particularly in those with hyposmia [5, 9]. In contrast, MIBG uptake remains largely unchanged in MSA and PSP, suggesting distinct mechanisms of autonomic dysfunction [8]. In MSA, severe early autonomic impairment is attributed to degeneration of autonomic centers, including the dorsal vagal nucleus in the brainstem, as well as parasympathetic nuclei, and sympathetic neurons in the spinal cord, which are less affected in PD [10]. PSP has less pronounced cardiovascular autonomic dysfunction, with in the disease neurogenic orthostatic hypotension being uncommon [4, 11], and predominant autonomic dysfunction being an exclusion criterion for diagnosis [2]. Differentiating Parkinsonian syndromes is challenging due to overlapping symptoms, particularly in the early stages. In iRBD, identifying converters to synucleinopathies is also crucial. Cost‐effective, accessible methods for assessing autonomic function could help diagnosis and disease stratification.

Heart rate variability (HRV), driven by the dynamic interaction of sympathetic and parasympathetic activity, serves as a marker of cardiac autonomic function [12]. It reflects beat‐to‐beat, or RR interval, fluctuations in heart rate, assessed via electrocardiogram (ECG), and is influenced by both internal and external factors [12]. The parasympathetic system induces immediate heart rate deceleration lasting one to two beats, whereas the sympathetic system causes a delayed, sustained acceleration lasting up to 30 s. Thus, short‐term, beat‐to‐beat variations are primarily parasympathetically mediated. Higher HRV reflects greater autonomic adaptability, while lower HRV suggests impaired autonomic regulation. HRV naturally declines with age, particularly due to reduced parasympathetic activity, and is influenced by factors such as sex, activity level, and pathological conditions, including myocardial infarction [13].

HRV analysis including the relatively newly identified DC [13, 14], covers time‐domain, frequency‐domain, and nonlinear parameters [12]. Time‐domain measures, such as the standard deviation of normal‐to‐normal intervals (SDNN) and the root mean square of successive differences (RMSSD), assess fluctuations between successive heartbeats. Frequency‐domain analysis, using Fourier transform, derives low‐frequency power (LF), reflecting both sympathetic and parasympathetic activity and baroreflex modulation; high‐frequency power (HF), linked to parasympathetic activity; and very low‐frequency power (VLF). Nonlinear measures analyze heart rate signals using complex mathematical models. The HRV triangular index (HRVI) is calculated using a geometric method as the total number of RR intervals divided by the maximum of the density distribution. This parameter reflects overall variability but is not directly linked to the sympathetic or parasympathetic autonomic nervous system [12, 15]. It is considered particularly resistant to artifacts [15]. In contrast, DC is derived from phase‐rectified signal averaging (PRSA) and quantifies the heart's ability to slow down and is a specific parasympathetic marker [14]. Unlike traditional HRV parameters, DC is relatively independent of respiratory influences, making it a robust prognostic marker in cardiology. Lower DC indicates reduced parasympathetic function and is associated with increased risks of heart failure, arrhythmias, and post‐myocardial infarction mortality, whereas higher DC reflects better autonomic regulation and lower cardiac risk [13]. Declaration capacity (DC) has shown greater predictive value for sudden cardiac death compared to conventional HRV measures [16] and may provide insights into autonomic dysfunction in parkinsonian syndromes. HRV has been studied in parkinsonian syndromes with mixed findings [17, 18]. Reduced HRV indices are consistently reported in early‐stage PD and have been linked to increased PD risk [18]. Similarly, decreased HRV is observed in iRBD, a major risk factor for PD and MSA [9]. Given its noninvasive nature and cost effectiveness, electrophysiological characterization appears to be a pragmatic approach for detecting autonomic dysfunction at various neurodegenerative stages. DC, though less studied in neurodegeneration, may be a valuable parameter for further autonomic investigation [19, 20, 21].

This pilot study investigates cardiac autonomic dysfunction in iRBD, PD, MSA, and PSP compared to healthy controls (HC). It explores whether DC could serve as a potential biomarker to differentiate atypical parkinsonian syndromes, such as MSA and PSP, from PD or help identify iRBD patients at risk of conversion to PD or MSA. Additionally, the study examines the relationship between DC and other HRV parameters.

## 2 Methods

This prospective pilot study conforms with the World Medical Association Declaration of Helsinki and approved by the ethics committee of the university of Marburg on December 30, 2012 (approval ID: 164/12). The study was conducted between January 2013 and October 2014 at the department of neurology, university hospital Marburg (UMR). The study adhered to the relevant STROBE checklist.

### 2.1 Study Participants and Clinical Assessments

Male and female patients aged 18–75 years were eligible if they provided written informed consent. Exclusion criteria included relevant cardiovascular diseases (e.g., history of myocardial infarction, heart failure NYHA ≥ II, advanced arrhythmias), and the use of medications on the examination day known to affect the autonomic nervous system, such as nitrates, alpha‐ and beta‐receptor agents, anticholinergics, cholinomimetics, and dopaminergic drugs. Additional exclusion criteria included medical interventions or surgeries affecting cardiac autonomic activity, including implanted pacemakers, bilateral cervical/thoracic sympathectomy, or cardiac surgery.

Patients were included in the following groups. (i) PD group: first PD diagnosis within ≤ 24 months based on Gelb et al. criteria, aligned with German Society for Neurology guidelines; Hoehn & Yahr stage ≤ 2; no clinical signs of MSA or PSP [22]; (ii) MSA group: clinically probable MSA per Gilman et al. criteria [3], without evidence of PD or PSP; (iii) PSP group: clinically probable PSP according to Litvan et al. criteria [23], with no evidence of PD or MSA; (iv) iRBD group: isolated RBD diagnosis per ICSD criteria [6], confirmed by video polysomnography, with no signs of PD, MSA, or PSP; (v) healthy control (HC) group: no evidence of RBD, PD, MSA, or PSP.

Clinical assessments included the Beck Depression Inventory (BDI), Montreal Cognitive Assessment (MoCA), Mini‐Mental State Test (MMST), Hoehn & Yahr staging (H & Y), REM sleep behavior disorder screening questionnaire (RBDSQ), Unified Parkinson's Disease Rating Scale (UPDRS), Parkinson's disease non‐motor symptoms scale (PD NMS), frontal assessment battery (FAB), progressive supranuclear Palsy progress score (PSPRS), and the Unified Multiple System Atrophy Rating Scale (UMSARS). Demographic data collected included sex, age, disease duration and body mass index (BMI).

### 2.2 Cardiovascular Autonomic Function Tests

Participants spent the night before ECG recording under controlled conditions, either in the hospital or, for HC and some iRBD patients, in a nearby hotel. On the examination day, they fasted for at least 12 h, abstaining from food, medications, and smoking, though drinking clear water was allowed. Testing was conducted in the morning between 7:30 and 10:00 a.m. under controlled wakeful conditions. After a 15‐min resting period in a darkened room, a 30‐min ECG recording was performed with participants in a supine position, elevated at 30°. Data were collected using a seven‐lead, three‐channel Holter ECG system (Getemed CardioMem CM 3000 SMA).

### 2.3 Heart Rate Variability (HRV) Data Analysis

ECG data, digitized at 128 Hz, were processed using CardioDay software under the supervision of Prof. Dr. Bauer. The software identified R‐peaks to determine RR intervals, as well as artifacts and extrasystoles. HRV parameters were analyzed according to the 1996 standards of the task force of the European society of cardiology and the North American society of pacing and electrophysiology [12]. The method for calculating DC and the related visual representations were based on the work described by Bauer et al. [14]. The analysis was conducted in a blinded manner. Additional parameters included mean heart rate (MHR) and the HRV parameters SDNN, RMSSD, LF, LF normalized units (LFn), HF, HF normalized units (HFn), very LF (VLF), and HRVI.

### 2.4 Statistical Analysis and Graphical Representation

Results of clinical assessments and demographic data were analyzed descriptively. HRV measurements and age were analyzed using nonparametric statistical tests in R software (version 3.2.0, R Core Team, 2015) [24]. A Kruskal–Wallis test was performed, followed by a post hoc Dunn‐Bonferroni test for pairwise comparisons. Grouped box plots were used for graphical representation. Statistical significance was set at *p* ≤ 0.05.

## 3 Results

This explorative study included 49 participants: 10 HC, ten iRBD patients, 10 PD patients, 10 MSA patients, and nine PSP patients.

### 3.1 Study of the Cohort Population

Table 1 summarizes the demographic data. Male participants were predominant across groups, accounting for 80% in the HC, iRBD, and PD groups, and 70% and 55% in the MSA and PSP groups, respectively. Mean age varied by up to 12 years, with MSA patients being the youngest (56 ± 7.1 years) and PSP patients the oldest (68 ± 7.9 years). Significant age differences were observed between PSP and MSA (*p* < 0.001), PSP and PD (58 ± 9.1 years) (*p* < 0.05), and MSA and HC (64 ± 5.3 years) (*p* < 0.05). No significant differences in BMI were found among groups. Results of clinical assessments, including mood, cognition, motor, and non‐motor symptom evaluations, are summarized in Table 2. Findings were consistent with disease‐specific characteristics.

**TABLE 1:** Sociodemographic characteristics and clinical assessment results of motor and non‐motor symptoms in study participants.

	HC (*n* = 10)	iRBD (*n* = 10)	PD (*n* = 10)	MSA (*n* = 10)	PSP (*n* = 9)
*Sociodemographic variables*					
Sex (M:F)	8:2	8:2	8:2	7:3	5:4
Age (years)	64 ± 5.3	63 ± 6.0	58 ± 9.1	56 ± 7.1^a^	68 ± 7.9^b^ ^,^ ^c^
Disease duration (years)	NA	4.9 ± 3.6	1.5 ± 0.3	3.9 ± 2.3	3.9 ± 1.7
BMI (kg/m^2^)	26.9 ± 3.7	26.2 ± 2.9	26.8 ± 4.86	28.9 ± 7.17	25.1 ± 7.9
*Clinical assessments*					
BDI	1.6 ± 1.6	8.9 ± 7.4	4.9 ± 4.7	14.5 ± 12.9	13.4 ± 5.7
MoCA	27.7 ± 1.8	28.2 ± 1.2	27.3 ± 1.6	26 ± 4.1	22.4 ± 3.8
MMST	29 ± 0.9	29.7 ± 0.5	29.7 ± 0.7	29.1 ± 1.5	27.5 ± 2.4
H & Y	NA	NA	1.2 ± 0.4	NA	NA
RBDSQ	0.6 ± 0.7	10.4 ± 1.5	3.9 ± 3.3	6.4 ± 3.3	2.3 ± 1.0
UPDRS total score	0.8 ± 1	6.9 ± 7.74	18.6 ± 10.8	61 ± 19.4	56 ± 25.6
UPDRS part I	0.5 ± 0.7	1.6 ± 1.6	0.3 ± 0.7	1.9 ± 1.2	3.4 ± 2.7
UPDRS part II	0.1 ± 0.5	2.3 ± 3.7	4.9 ± 3.3	22 ± 9.6	21 ± 9.3
UPDRS part III	0.2 ± 0.5	2.4 ± 3.2	12.5 ± 6.8	39.7 ± 14.9	31.4 ± 15.3
PDNMS	1.9 ± 1.1	9.3 ± 3.6	5.6 ± 4.0	11.5 ± 3.3	11.78 ± 2.4
FAB	18 ± 7.9	NA	NA	NA	12 ± 2.8
PSPRS	0.6 ± 7.9	NA	NA	NA	42 ± 12.0
UMSARS part I and II	1 ± 1.4	NA	NA	48.1 ± 8.3	NA
UMSARS part I	0.8 ± 1.3	NA	NA	22.7 ± 8.3	NA
UMSARS part II	0.1 ± 0.3	NA	NA	24.5 ± 9.4	NA
UMSARS part III impaired N %	0	NA	NA	50	NA
UMSARS part IV	0.9 ± 10.3	NA	NA	3 ± 1.2	NA

*Note:* Values are expressed as mean ± standard deviation.

Abbreviations: BMI, body mass index; F, female; HC, healthy control group; iRBD, isolated REM sleep behavior disorder group; M, male; NA, not applicable; MSA, multiple system atrophy group; PD, Parkinson's disease group; PSP, progressive supranuclear palsy group; y, years. Clinical assessments: BDI, Beck depression inventory; FAB, frontal assessment battery; PSPRS, progressive supranuclear Palsy progress score; H & Y, Hoehn &Yahr stage; MMST, mini‐mental state test; MoCA, montreal cognitive assessment; NA, not applicable; PD NMS, Parkinson's disease non‐motor symptoms scale; RBDSQ, REM sleep behavior disorder screening questionnaire; UMSARS, unified multiple system atrophy rating scale; UPDRS, unified Parkinson's disease rating scale.

^a^ *p* < 0.05 compared to HC.

^b^ *p* < 0.05 compared to PD.

^c^ *p* < 0.01 compared to MSA.

**TABLE 2:** Clinical assessment results of motor and non‐motor symptoms in study participants.

	HC (*n* = 10)	iRBD (*n* = 10)	PD (*n* = 10)	MSA (*n* = 10)	PSP (*n* = 9)	
*HRV parameter*						
MHR	60.59 ± 6.65	61.10 ± 16.20	63.92 ± 15.48	65.41 ± 10.13	74.34 ± 14.06	0.09
SDNN	57.93 ± 24.31	67.75 ± 77.17	86.23 ± 88.74	43.94 ± 18.66	40.20 ± 21.85	0.42
RMSSD	39.61 ± 18.98	92.41 ± 147.55	109.33 ± 179.81	46.68 ± 27.79	40.08 ± 23.03	0.94
HF	234.38 ± 223.36	660.31 ± 978.70	4217.72 ± 9114.41	270.76 ± 323.43	371.98 ± 402.21	0.87
HFn	0.24 ± 0.17	0.38 ± 0.2	0.46 ± 0.28	0.41 ± 0.12	0.39 ± 0.17	0.21
LF	1406.81 ± 1752.36	1909.4 ± 5142.02	1049.65 ± 1666.09	237.44 ± 356.28	290.66 ± 401.16	0.07
LFn	0.73 ± 0.23	0.58 ± 0.v23	0.50 ± 0.31	0.45 ± 0.21	0.44 ± 0.26	0.08
VLF	1,015,314 ± 212,346	982,462 ± 469,690	1,046,262 ± 522,396	907,442 ± 314,050	727,535 ± 285,248	0.22
HRVI	29.08 ± 14.07	17.92 ± 7.67	24.77 ± 16.93	19.89 ± 5.70	13.8 ± 5.43^a^	0.04
DC	9.66 ± 4.67	5.85 ± 2.43	7.55 ± 2.48	3.82 ± 1.38^a^	3.19 ± 2.77^a^, ^b^	< 0.001

*Note:* A Kruskal–Wallis test was performed, followed by a post hoc Dunn–Bonferroni test for pairwise comparisons. Values are expressed as mean ± standard deviation.

Abbreviations: DC, deceleration capacity; HC, healthy control group; HF, high‐frequency power; HFn, normalized HF; HRVI, heart rate variability index; iRBD, isolated REM sleep behavior disorder group; LF, low‐frequency power; LFn, normalized LF; MHR, mean heart rate per minute; MSA, multiple system atrophy group; PD, Parkinson's disease group; PSP, progressive supranuclear palsy group; RMSSD, root mean square of successive differences; SDNN, standard deviation of normal‐to‐normal intervals; VLF, very low frequency power.

^a^ *p* < 0.05 compared to HC.

^b^ *p* < 0.05 compared to PD.

### 3.2 Results of Heart Rate Variability (HRV) Parameters

Besides MHR, HRV parameters included SDNN, RMSSD, HF, HFn, LF, LFn, VLF, HRVI, and DC (Table 1). Statistically significant group differences were found for DC and HRVI, whereas all other cardiac parameters showed no significant differences between groups. Given the small sample size and the considerable interindividual variability, as reflected by large SD, some variables such as HF, an absolute non‐normalized measure, show marked differences between groups without any statistical significance or trend.

**FIGURE 1:**
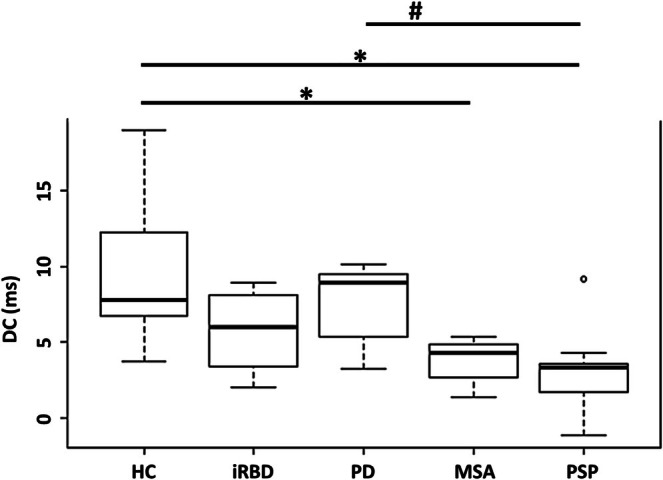
Boxplots of deceleration capacity (DC) across disease groups. Boxplots of deceleration capacity (DC). The *Y*‐axis represents absolute values: DC in milliseconds. Black line: Median. Box: Interquartile range (middle 50% of the data). Whiskers: 95% of the data distribution. Circles: Subjects with values exceeding 1.5 times the interquartile range. HC, healthy control group; iRBD, isolated REM sleep behavior disorder group; MSA, multiple system atrophy group; PD, Parkinson's disease group; PSP, progressive supranuclear palsy group. *p < 0.05 compared to HC. # *p* < 0.05 compared to PD.

Mean DC values were, as expected, highest in HC (9.66 ± 4.67), followed by PD patients (7.55 ± 2.48), which were unexpectedly higher than those in iRBD patients (5.85 ± 2.43). No significant differences or trends were observed in DC between patients with PD and those with iRBD. As expected, MSA patients had low DC values (3.82 ± 1.38), while PSP patients had even lower values (3.19 ± 2.77). Significant group differences in DC were observed between HC and PSP (*p* = 0.003) and between HC and MSA (*p* = 0.010). Additionally, DC was significantly lower in PSP than in PD (*p* = 0.025). A trend toward significance was observed for iRBD compared to HC (*p* = 0.068) and for PD compared to MSA (*p* = 0.069) (Figure 1). These differences between groups remained significant (*p* < 0.05) after adjustment for age and disease duration. PD patients have DC values very close to HC, who present a much wider distribution of values. This explains the paradox of a slightly higher median value in PD patients as compared to the HC (Figure 1, Table 1). Two patients with PD and one patient with iRBD had a history of beta‐blocker use. The PD patients on beta‐blockers had DC values in the lower third compared to the PD group (mean 7.55 ± 2.48), with values of 5.34 and 5.22. In contrast, the iRBD patient had a DC of 8.88 which was in the upper third compared to the mean for iRBD patients (5.85 ± 2.43). A potential rebound effect of sympathetic activity should still be considered. HRVI was significantly reduced in PSP patients compared to HC (*p* = 0.012). Healthy controls (HC) participants had the highest HRVI values (29.08 ± 14.07), while PSP patients had the lowest (13.8 ± 5.43). The HRVI values of iRBD patients (17.92 ± 7.67) were lower than those of PD (24.77 ± 16.93) and MSA (19.89 ± 5.7) patients.

## 4 Discussion

This exploratory study found significantly lower DC values in PSP and MSA patients compared to HC, indicating cardiac autonomic dysfunction. Unexpectedly, the reduction was more pronounced in PSP than in MSA. While DC values in PD patients were significantly different from those in PSP, they only showed a trend toward differentiation from MSA. Additionally, PSP patients had a significant decrease in HRVI, reflecting an overall reduction in HRV.

### 4.1 Limitations and Strengths of the Study

This well‐controlled pilot study included not only HC but also prodromal aSYN patients with iRBD, as well as patients with PD, MSA, and PSP. All participants were recorded under highly standardized conditions, following strict inclusion and exclusion criteria, and after overnight fasting with the last medication intake the evening before the recordings. In addition to DC, other time‐ and frequency‐domain parameters were also assessed.

Despite these strengths, the study has several limitations. The small sample size and group heterogeneity likely have influenced the results. Due to the rarity of atypical parkinsonian syndromes, this pilot study included limited group sizes of suitable patients, with the PSP group slightly underrepresented (nine instead of ten patients). Furthermore, clinical subtypes of PD, MSA, or PSP were not differentiated, which may have impacted the results. In addition, as HRV declines with age [25], our findings are limited by significant age differences between groups, particularly between HC participants and PSP/MSA patients, as well as between PSP and PD patients [12]. This could lead to an overestimation of autonomic dysfunction in PSP patients and an underestimation in MSA patients. Moreover, the absence of universally comparable age‐dependent reference values limits the interpretability of absolute measurements [26]. Other potential confounders, such as physical fitness, breathing rate, hormone levels, and circadian rhythm, were not assessed [12]. Comorbidities such as diabetes mellitus and peripheral neuropathy, which met the inclusion criteria, were not specifically accounted for in the analysis. Additionally, detailed information on medications, such as beta‐blockers, in the days preceding the examination was not collected, which may have exerted a rebound effect. HRV differences have been observed in the akinetic‐rigid PD subtype, which exhibits reduced LF, indicative of impaired baroreflex modulation [18, 27]. Prior research suggests minimal differences in cardiac autonomic dysfunction in MSA subtypes [27]. The iRBD group may also be heterogeneous, as patients may progress to PD, dementia with Lewy bodies (DLB), or, rarely, MSA [6]. Another limitation is the lack of histopathological confirmation of the diagnosis in the enrolled patients. Additionally, only early‐stage PD patients were included to better compare the iRBD and PD groups, which does not fully represent the entire spectrum of autonomic nervous system (ANS) dysfunction in PD.

In cardiology, HRV analysis follows established guidelines for measurement, interpretation, and clinical use [12]. However, no standardized recommendations exist for patients with neurodegenerative diseases, leading to varied approaches. Studies on HRV in parkinsonian syndromes and iRBD show inconsistencies due to differing parameters and insufficiently standardized conditions, sometimes resulting in contradictory interpretations. HRV, including DC, is a recognized marker of cardiac autonomic dysfunction [14], requiring standardized yet practical data collection. A 30‐min recording captures all standard HRV parameters except ultra LF (ULF) and is clinically feasible. Autonomic function varies with circadian rhythms, wakefulness, and sleep. Although recordings were conducted under controlled conditions, the absence of EEG data means that relaxation, somnolence, or possibly sleep may have influenced DC modulation. External factors like physical activity, stress, and emotions also affect ANS function. To minimize these effects, measurements were taken at rest, with patients abstaining from food and medication on the examination day. However, individual responses to relaxation, stress, or fasting may have introduced variability.

### 4.2 Deceleration Capacity (DC) as a Vagal Measure and Interpretation of Heart Rate Variability (HRV)

Cardiac sympathetic innervation is commonly assessed via MIBG scintigraphy, but no routine imaging method exists for visualizing parasympathetic innervation. Alongside established parasympathetic HRV parameters like SDNN and HF, DC is a relatively novel marker of autonomic cardiac modulation, particularly parasympathetic function [14]. Its role in parkinsonian syndromes remains poorly described [19, 20, 21]. In our data, DC was significantly reduced in patients with atypical parkinsonian syndromes, yet this was not reflected in other parasympathetic markers such as HF and RMSSD. One possible explanation is the high interindividual variability of HRV [28], which is especially relevant in small cohorts. This raises the question of whether DC may be more sensitive than conventional HRV parameters, warranting further investigation in larger studies. Notably, Bauer et al. identified DC as a prognostic marker for post‐myocardial infarction mortality, with reductions in DC and overall HRV linked to increased mortality [13, 16]. Given these findings, patients with PSP or MSA may benefit from cardiological assessment and follow‐up to determine potential implications for their prognosis.

### 4.3 Heart Rate Variability (HRV) and Cardiovascular Dysautonomia in PSP, MSA, PD, and iRBD: Insights From the Literature

Previous reports of cardiovascular autonomic dysfunction in PSP have not described severe impairment and suggested a parasympathetic origin, aligning with our findings [11]. Cardiovascular symptoms, particularly orthostatic complaints, have been reported in up to 50% of PSP patients based on questionnaires or clinical interviews [11]. However, these subjective symptoms seem not to correlate with objective autonomic testing, and there are no evident signs of neurogenic orthostatic hypotension in PSP [4, 11]. This discrepancy may indicate that standard autonomic tests lack the sensitivity to detect the underlying pathophysiological mechanisms responsible for these symptoms. In MSA patients, cardiovascular autonomic dysfunction is a key non‐motor feature from early disease stages on [10, 21]. While cardiovascular dysfunction in PSP is primarily parasympathetically driven, MSA studies report mixed findings. Some attribute the dysfunction predominantly to preganglionic sympathetic denervation, whereas others suggest parasympathetic impairment attributed to neuronal degeneration in the ventrolateral nucleus ambiguus [10, 17]. HRV parameters, particularly DC, as suggested by a trend observed in the present study, may aid in differentiating MSA from PD [18, 21].

In PD, parasympathetic denervation initially affects the gastrointestinal tract, while the cardiovascular system primarily undergoes sympathetic denervation [24]. Sympathetic HRV parameters are significantly reduced in early PD, whereas cardiac parasympathetic innervation remains largely intact [7]. Lower HRV correlates with the severity of motor symptom [17]. Additionally, PD patients with symptomatic RBD show greater HRV impairment than those without RBD [29]. Since dysautonomia can emerge in prodromal PD, HRV, particularly sympathetic parameters, may serve as an early biomarker [7]. The tendency toward reduced DC in our iRBD patients compared to PD, rather than the expected intermediate profile between HC and PD, may reflect a very early disease stage in our PD group. Most studies report HRV markers as intermediate between HC and PD, supporting the concept of progression from a preclinical to a neurodegenerative stage. HRV findings in iRBD suggest both sympathetic and parasympathetic cardiac dysfunction [17]. However, as MSA is rare, no HRV data exist for iRBD patients who later convert specifically to MSA. Several studies link reduced HRV parameters to an increased risk of PD [18]. Interestingly, significant attenuation of parasympathetic HF values during all sleep stages has been observed in RBD, whether isolated or symptomatic of PD, compared to PD without RBD [30].

## 5 Conclusion

The significant reduction in DC in PSP and MSA suggests pronounced cardiac parasympathetic dysfunction. While we confirmed the reduced DC values in MSA, this finding is novel for PSP. DC is a promising noninvasive tool for assessing ANS regulation in synucleinopathies, but larger studies are needed to validate its role as a screening marker, accounting for subtypes, comorbidities, and age‐matched controls. Longitudinal assessments, particularly in iRBD, could clarify the relation between early autonomic dysfunction and disease progression, while HRV measurements during sleep may provide a more standardized evaluation, as sleep stages modulate autonomic activity. Despite limitations, DC may help differentiate PD from atypical parkinsonian syndromes like MSA and PSP, which remain challenging to diagnose early. Given the impact of autonomic dysfunction on quality of life and mortality, comprehensive autonomic testing should be part of the diagnostic workup.

## Author Contributions

Conceptualization: W.H.O., D.V., V.R., U.K.; data curation: N.M., D.V., E.S., K.K., A.B.; formal analysis: A.B., K.K., D.V., N.M.; investigation: W.H.O., V.R.; methodology: D.V., V.R., W.H.O., K.K., A.B.; supervision: V.R., W.H.O.; validation: A.B., K.K., W.H.O.; visualization: K.K., A.B., N.M.; writing – original draft: E.R.; writing – review and editing: W.H.O., A.J. All authors have approved the final article.

## Conflicts of Interest

The authors declare no conflicts of interest.

## Data Availability

The data that support the findings of this study are available from the corresponding author upon reasonable request.
